# Multi-Season Phenology Mapping of Nile Delta Croplands Using Time Series of Sentinel-2 and Landsat 8 Green LAI

**DOI:** 10.3390/rs14081812

**Published:** 2022-04-09

**Authors:** Eatidal Amin, Santiago Belda, Luca Pipia, Zoltan Szantoi, Ahmed El Baroudy, José Moreno, Jochem Verrelst

**Affiliations:** 1 Image Processing Laboratory (IPL), University of Valencia, Catedrático Agustín Escardino 9, 46980 Valencia, Spain; 2 Department of Applied Mathematics, University of Alicante, 03690 Alicante, Spain; 3 Institut Cartogràfic i Geològic de Catalunya (ICGC), Parc de Montjüic, 08038 Barcelona, Spain; 4 Science, Applications & Climate Department, European Space Agency, 00044 Frascati, Italy; 5 Department of Geography & Environmental Studies, Stellenbosch University, 7602 Stellenbosch, South Africa; 6 Faculty of Agriculture, Tanta University, 31527 Tanta, Egypt

**Keywords:** green leaf area index, Sentinel-2, Landsat 8, land surface phenology, Gaussian process regression (GPR), time series analysis

## Abstract

Space-based cropland phenology monitoring substantially assists agricultural managing practices and plays an important role in crop yield predictions. Multitemporal satellite observations allow analyzing vegetation seasonal dynamics over large areas by using vegetation indices or by deriving biophysical variables. The Nile Delta represents about half of all agricultural lands of Egypt. In this region, intensifying farming systems are predominant and multi-cropping rotations schemes are increasing, requiring a high temporal and spatial resolution monitoring for capturing successive crop growth cycles. This study presents a workflow for cropland phenology characterization and mapping based on time series of green Leaf Area Index (LAI) generated from NASA’s Harmonized Landsat 8 (L8) and Sentinel-2 (S2) surface reflectance dataset from 2016 to 2019. LAI time series were processed for each satellite dataset, which were used separately and combined to identify seasonal dynamics for a selection of crop types (wheat, clover, maize and rice). For the combination of L8 with S2 LAI products, we proposed two time series smoothing and fitting methods: (1) the Savitzky–Golay (SG) filter and (2) the Gaussian Processes Regression (GPR) fitting function. Single-sensor and L8-S2 combined LAI time series were used for the calculation of key crop Land Surface Phenology (LSP) metrics (start of season, end of season, length of season), whereby the detection of cropland growing seasons was based on two established threshold methods, i.e., a seasonal or a relative amplitude value. Overall, the developed phenology extraction scheme enabled identifying up to two successive crop cycles within a year, with a superior performance observed for the seasonal than for the relative threshold method, in terms of consistency and cropland season detection capability. Differences between the time series collections were analyzed by comparing the phenology metrics per crop type and year. Results suggest that L8-S2 combined LAI data streams with GPR led to a more precise detection of the start and end of growing seasons for most crop types, reaching an overall detection of 74% over the total planted crops versus 69% with S2 and 63% with L8 alone. Finally, the phenology mapping allowed us to evaluate the spatial and temporal evolution of the croplands over the agroecosystem in the Nile Delta.

## 1 Introduction

Satellite Earth observation data are increasingly used for analyzing dynamic land surface processes at different spatial and temporal scales [1–3]. Their processing into easy interpretable products become a valuable tool in relevant contexts, such as food security, assisting agricultural practices or studying the vegetation response to environmental changes [4–6]. Current agriculture challenges are related to the growing global population aiming to yield increase and sustainable intensification of cropland production [7–9], which requires regular crop growth monitoring in agroecosystems. Based on satellite data, changes in vegetation dynamics and crop growth evolution can be studied through the estimation of Land Surface Phenology (LSP) metrics from vegetation indices or biophysical variables time series [10]. Among the LSP metrics relevant for croplands, the most important ones are start-of-season (SOS) and end-of-season (EOS), marking the green-up and senescing of the crop growth cycle [11]. LSP products have been traditionally developed from coarse to moderate spatial resolution satellites, such as Moderate Resolution Imaging Spectroradiometer (MODIS), Advanced Very High Resolution Radiometer (AVHRR) and Visible Infrared Imaging Radiometer Suite (VIIRS) [12–17]. Thanks to the frequent image acquisitions of these missions, derived LSP products allowed regional to global long-term phenology detection of vegetation across diverse land cover types, but with low spatial detail and with the occurrence of mixed pixels over heterogeneous landscapes. For cropland growth cycle monitoring, however, both fine spatial and temporal resolutions are required to enable characterizing individual crop fields at their different phenological stages [18,19]. Available imagery from Landsat 8 (L8) at 30 m, with a single orbit and a 16-day repeat cycle, favoured time series and data fusion studies for identifying phenology patterns at the field scale [20–24]. On the other hand, ESA’s Sentinel-2 (S2) constellation (S2A/S2B) provides imagery at an even shorter revisit frequency, up to 5 days, and with similar spatial and spectral characteristics as L8. The combination of both satellites, therefore, provides optimal systematic land imaging coverage adequate for timely cropland mapping applications [25]. NASA’s initiative to create the Harmonized Landsat and Sentinel-2 (HLS) dataset brings forth freely available time series of spectral and radiometric consistent surface reflectance imagery with a uniform gridding from both satellites [26]. The HLS dataset has already been used in vegetation monitoring studies, showing a good performance and sufficient capability to capture seasonal dynamics of vegetation phenology, with high agreement compared to coarse-resolution satellites estimations, but at a finer spatial resolution of 30 m [21,27]. The HLS dataset was also positively evaluated in its feasible shorter revisit time to monitor grasslands [28], and proved to be able to achieve high overall accuracies of cropland mapping over different regions across the world [29]. Hence, the HLS dataset emerged as a potentially ideal data source for cropland phenology monitoring.

When it comes to LSP metrics estimation, widely used products derived from satellite data streams include spectral bands combination of vegetation indices (VIs) and biophysical variables obtained from either physical or empirical retrieval models [10]. Similarly to the broadly used VIs, i.e., normalized difference vegetation index (NDVI), enhanced vegetation index (EVI) and two-band EVI (EVI2) [11], which used alone can present a limited sensitivity to changes of vegetation photosynthesis dynamics [30] and high biomass growth stages [31,32], biophysical variables such as Leaf Area Index (LAI) can reflect the seasonal evolution of photosynthetic active vegetation in addition to serve as indicator of canopy structure condition [33]. Moreover, while established VIs represent an arithmetic formulation based on a few bands to maximize the spectral response to vegetative components, an LAI multiband retrieval model can exploit the full spectral information available from the satellite [34–36]. LAI is an essential input for land surface and atmospheric energy exchange modeling [37] and has been used for global scale phenology monitoring as well as to map cropping systems [38,39]. Despite the lower proportion of LAI-based phenology studies published, it was previously shown as a biophysical variable capable to improve the detection performance over croplands with respect to the EVI [40].

The timing of cropland phenology seasonality and growing seasons length are typically estimated as LSP metrics corresponding to key dates of the crop growth cycle by time series processing and fitting functions approaches [41–43]. As pre-processing for later extraction of LSP metrics, data smoothing methods are usually applied in order to reduce time series disturbances and possible artifacts [44,45]. Established methods include double logistic and asymmetric Gaussian functions and the moving Savitzky–Golay filter for noise mitigation of remote sensing time series [10]. More recently, machine learning fitting techniques emerged as attractive alternatives for bringing the possibility of further perform time series gap-filling with promising results [46]. In particular, the Gaussian Processes Regression (GPR) algorithm proved to be a competitive gap-filling technique, being able to additionally provide an approximation of the estimates uncertainty [47,48].

Compared to other vegetation canopies, time series analysis over croplands can be particularly challenging due to highly dynamic seasonal growth cycles, management practices and the complex spatial patterns in areas with high cropping frequency, with more than a single growing season per year [49]. LSP metrics calculation allow to map the cropland extent and characterize seasonality of different crop types. The simplest way to estimate cropping frequency is by counting the number of growing seasons as the number of peaks corresponding to a complete growth cycle in the time series [12,50]; while a single cropping time series presents one growth cycle per year, the identification of two to three crop cycles within a single year is more difficult. Moreover, multi-cropping patterns can span over consecutive years with different crop types rotations [22,51]. Standard phenology detection methods essentially extract transition dates representative of crop growth stages from the time series curve, either based on identifying curvature changes or defining a magnitude thresholds [44,46]. Particularly, threshold-based methods are generally adapted by the user accounting for the variability of cropland seasonal dynamics of the study site by setting temporal constraints and maximum magnitude thresholds to dismiss noisy peaks or minimize the non-vegetated spectral influence [10].

Altogether, the aim of this study is to demonstrate the added value of using harmonized S2 and L8 data streams, i.e., HLS dataset, with respect to single-sensor based approaches for characterizing the phenology of multi-cropping systems. To this end, we present a workflow using green LAI time series derived from the HLS data stream. The following objectives were defined: (1) to adapt an S2 GPR retrieval model to L8 imagery in order to obtain consistent estimations from both sensors data; (2) to derive, respectively, distinct green LAI time series and subsequently combine them; (3) to extract phenology indicators of different crop types and assess the consistency of the crop seasonal dynamics retrieved from different time series datasets during the same time period; and (4) to generate final crop phenology descriptor maps. The remainder of this paper is structured as follows. Section 2 describes the generation process of green LAI time series used for LSP metrics estimation. Section 3 presents the results of crop phenology characterization and mapping, while major findings and limitations of this work are discussed in Section 4. Finally, conclusions and future research lines are provided in Section 5.

## 2 Materials and Methods

**Figure 1:**
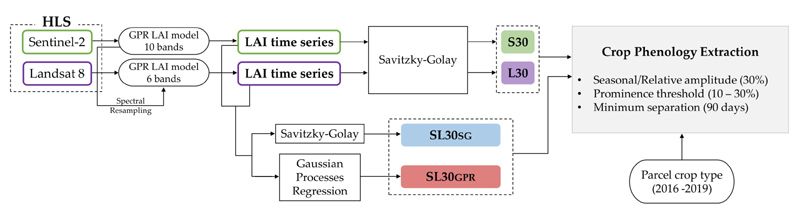
Workflow of the proposed methodology for cropland phenology extraction from HLS.

With the ambition to characterize multi-season cropland phenology with HLS data [26], we propose the workflow sketched in Figure 1. First, HLS tile time series were separately processed for green LAI estimation applying a retrieval model originally developed for S2 [52], and here adapted to the spectral configuration of L8. As such, we obtained two single-sensor green LAI time series, which were separately processed with a Savitzky–Golay (SG) [53] smoothing filter based on a local average moving window. Second, both single-sensor LAI data streams were combined using two time series processing methods: (1) SG and (2) Gaussian Process Regression (GPR) [54], a machine learning fitting technique able to perform temporal gap-filling interpolation [48]. Next, the four green LAI data collections generated were analyzed on their ability to identify multi-season growth cycles by extracting a set of key LSP metrics (start of season, end of season, length of season) per crop type at parcel level. To do so, two different threshold strategies were applied defining either a seasonal or a relative amplitude value [46,55]. The performances of both methods were assessed by quantifying the number of growth seasons detected per crop type during the period of time studied, and then compared against crop calendar data.

Finally, the phenology extraction process was applied to the combined GPR LAI time series for mapping the annual evolution of cropland phenology and cropping frequency over the study site. The complete data processing chain and analysis are detailed in the following sections.

### 2.1 Study Area

The Nile Delta accounts for nearly half of all agricultural lands of Egypt. This region is characterized by an arid climate, with relatively moderate temperatures and low precipitation occurring mostly during winter. Annual rainfall ranges from 100 to 150 mm and annual mean temperature is around 21.5 °C. A variety of crop types are cultivated by irrigation with water supply coming from the Nile. Major summer crops are rice and maize. Winter wheat is the third most important cash crop in terms of production area, while the Egyptian clover is a major winter forage crop. These crops are alternatively cultivated, allowing to identify multiple crop cycles within a single year. Extended perennials annual crops include grape and citrus species. The absence of remarkable variations in climate conditions allow to establish a reference crop calendar, with regular plant and harvest dates (Table 1). It should be noted that these are the dates usually employed for crop management over all years, and here provide a reference framework to enable evaluation of the identified crop phenology evolution.

**Figure 2:**
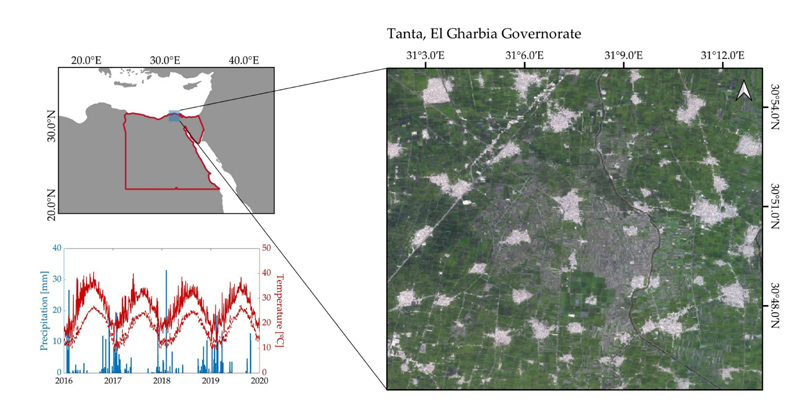
Location and climatology of the study site over the region of Tanta (Egypt).

Information on crop type planting was collected from 2016 to 2019 within the study site (Figure 2), which is an agricultural area covering approximately 326 ha in the region of Tanta, within El Gharbia Governorate. From 162 parcels available in total, 55 are under crop rotation schemes with more than 1 crop type per year, planted and harvested twice annually. The remaining 107 parcels correspond to single-cropping systems with the same annual crop type. We focus on major cash crops, namely, rice, maize, wheat and clover, excluding fruit and tree crops. Parcels’ delimitation into a geospatial vector file for carrying out the later processing per parcel crop type was provided by the University of Tanta (Egypt).

### 2.2 Harmonized Landsat and Sentinel-2 (HLS)

NASA’s Harmonized Landsat and Sentinel-2 (HLS) surface reflectance imagery data streams were used to conduct this study. The HLS dataset is routinely produced, over a limited number of regions across the world, from observations of the Operational Land Imager (OLI) aboard Landsat 8 (L8) and the Multi-spectral Instrument (MSI) aboard Sentinel-2A/B (S2), providing seamless products from both sensors, based on applying a common atmospheric correction algorithm [56] and performing radiometric and geometric adjustments [26]. HLS provides separately BRDF-normalized surface reflectance, respectively, derived from MSI and OLI, using a fixed solar angle and a common nadir view. For the bands common to both sensors, the MSI spectral bands are adjusted to OLI’s spectral bandpasses, which are used as reference. The HLS projection and gridding correspond to the tilling system used by Sentinel-2, which is UTM/WGS84 projection [26]. We used the current version of the HLS dataset (v1.4) corresponding to tile 36RUV (T36RUV) at 30 m resolution, from the beginning of 2016 to the end of 2020. Only images with less than 50% of cloud coverage were selected to further processing. This led to a collection of 221 S2 images and 173 L8 images.

### 2.3 Green LAI Retrieval

The HLS L8 and S2 datasets were separately processed into independent data streams of green LAI based on an earlier S2 green LAI model that was developed within the framework of the H2020 project SENSAGRI (Sentinels Synergy for Agriculture (http://sensagri.eu/, accessed on 24 January 2022) [52]. Gaussian Processes Regression (GPR) was chosen as retrieval modeling technique as a competitive machine learning regression algorithm in vegetation properties mapping applications in terms of prediction accuracy [36,57], and noteworthy for providing uncertainty estimates of the predictions [58], as well as insight on the bands relevance in the trained model [59]. In brief, a GPR model was trained from data pairs of green LAI ground-based measurements and their corresponding S2 reflectance spectra, which was either synthesized from hyperspectral data or extracted from simultaneous S2 imagery, covering the S2 spectral bands of 10 and 20 m spatial resolution, i.e., 10 bands in total [60]. The training dataset comprises a variety of crop types over diverse agricultural sites across Europe. Additionally, training samples of forested and non-vegetation areas were added to improve the performance of the retrieval model and to make the model widely applicable. A total of 218 data samples were used to train the green LAI retrieval model, which was validated based on an independent dataset (R^2^ = 0.7, RMSE = 0.67 m^2^/m^2^). Detailed information about data sampling and validation procedures can be found in [52]. Given the spectral configuration compatibility between S2 and L8 (Table 2), a new model adapted to L8 was developed. The spectral data of the described training dataset were resampled according to the band settings of L8; 6 bands ranging from the visible to the shortwave infrared (SWIR). Subsequently, these data were used to train a new GPR model. A general introduction into GPR can be found in [61], and a summary of the generic formulation is given in the next section.

### 2.4 Gaussian Processes Regression

In general, the GPR technique models the relation between input samples **x** ∈ R^*D*^ and output observations y∈ℝ as y=f(x)+ϵ, where *ϵ* is an additive Gaussian noise with zero mean and variance $\sigma_{n}^{2}$, and *f* (**x**) is a Gaussian-distributed random vector with zero-mean and covariance matrix **K**(**x**, **x**), i.e., *f*(x) ∼ 𝒩(0, **K**). The role of the Covariance matrix is to encode the similarity between each combination of the input samples **x**_*i*_ and **x**_*j*_ using a kernel function *k*(**x**_*i*_, **x**_*j*_). The covariance design is of paramount relevance, as it must take into account the main properties of the variable to be modeled. Concerning the retrieval of vegetation properties such as LAI from Earth observation data, the asymmetric Square Exponential (SE) kernel is usually preferred due to its capability to (1) successfully approximate smoothly varying functions and (2) take into account asymmetries in the feature space [54].

The asymmetric SE defines the covariance kernel function as:

(1) $$k(x_{i},x_{j})=\sigma_{s}^{2}\exp\left(-\frac{1}{2}\sum_{b=1}^{D}{\left[\frac{x_{i}(b)-x_{j}(b)}{\sigma_{b}}\right]}^{2}\right),$$

where $\sigma_{s}^{2}>0$ represents the output variance and *σ_b_* is related to the spread of the training information along the input dimension *b* in a way that the inverse of *σ_b_* describes the relevance of band *b* in the prediction process: the higher *σ_b_* the lower informative content of *b*. The covariance matrix is completely defined once the kernel’s free parameters and the noise variance $\sigma_{n}^{2}$ are set. These terms, usually referred to as GPR model’s hyperparameters, can be collectively denoted as $\theta =\{\sigma_{s}^{2},\sigma^{2},\sigma_{n}^{2}\}$, where $\sigma =\{\sigma_{1},\cdots ,\sigma_{D}\}$.

The Bayesian framework of GPR allows estimating the distribution of *f* at any test point *x*_∗_ conditioned on the information carried by the training data. According to its formulation, *f* (*x*_∗_) is normally distributed with mean and variance given by:

(2) $$\begin{array}{l} f(x_{*})=k_{*}^{T}{\left(k+\sigma_{n}^{2}I_{N}\right)}^{-1}y\\ \sigma_{f}^{2}(x_{*})=c_{*}-k_{*}^{T}{\left(K+\sigma_{n}^{2}I_{N}\right)}^{-1}k_{*} \end{array}$$

where *N* is the number of training samples, $k_{*}={\left[k(x_{*},x_{1}),\ldots ,k(x_{*},x_{N})\right]}^{T}$ is an *N* × 1 vector containing the similarity between ***x***∗ and the training input information, ***y*** = [*y*_1_, …, *y_N_*]^*T*^ is the training output and $c_{*}=k(x_{*},x_{*})+\sigma_{n}^{2}$.

The probability of the observations given the model’s hyperparameters p(y|x,θ) is provided by the marginal likelihood over the function values *f* [61], whose logarithmic expression is:

(3) $$\log p(y|x,f)=-\frac{1}{2}y^{T}{\left(K+\sigma_{n}^{2}I_{N}\right)}^{-1}y-\frac{1}{2}\log|k+\sigma_{n}^{2}I_{N}|-\frac{n}{2}\log2\pi$$

Equation (3) is made up of three terms: the first one is essentially a data-fit term, the second one represents a complexity penalty and the last one is just a normalizing constant. The maximization of the marginal likelihood, i.e., the minimization of Equation (3), provides directly the optimum value of ***θ***. This optimization procedure is usually referred to as training the GPR [61,62]. Once ***θ*** values have been estimated, the prediction of *y* for a new input vector ***x***∗ is given along with its uncertainty by Equation (2).

Finally, the formulation for the time series domain is straightforward. By imposing the dimension of input data be unitary, i.e., *D* = 1, and substituting **x** for *t*, the hyperparameters of the GPR model to be employed to gapfill the collection of a generic surface property *P_S_* such as LAI become $\theta_{t}=\{\sigma_{st}^{2},\sigma_{t}^{2},\sigma_{snt}^{2}\}$, and the corresponding SE kernel for covariance estimation is given by:

(4) $$k_{t}(t_{i},t_{j})=\sigma_{st}^{2}\exp\left(-\frac{1}{2}{\left[\frac{t_{i}-t_{j}}{\sigma_{t}}\right]}^{2}\right),$$

where *t_i_* and *t_j_* denotes two generic acquisition dates. The estimation of LAI at any dates is still given by Equation (2), with the new covariance matrix **K_t_** being now calculated using the optimized ***θ_t_*** among the elements of the input time series.

### 2.5 Smoothing and Gap-Filling

Smoothing filters allow to mitigate the noise effect to produce enhanced time series for later feature extraction [10,44]. In particular, the Savitzky–Golay (SG) filter minimizes the least-squares error in fitting a polynomial function to noisy data within a moving window centered at a point [53]. In this study, the smoothing window size (span) was set to 7 and the polynomial function to a quadratic. The resulting smoothed value (*g_i_*) is obtained from a linear combination according to the following expression:

(5) $$g_{i}=\frac{\sum_{n=-nL}^{nR}c_{n}f_{i+n}}{n}$$

where *f_i_* represents the original data point value, *n* is the width of the moving window and *nL* and *nR* are the left and right edges of the defined window frame. Then, each data value is replaced by a locally weighted average of nearby data points, and thus, the greater the value of the span is, the smoother is the fitted curve. SG is considered essentially a low-pass filtering method, which tends to preserve high frequency signal components, and responds less effectively in case of high noise levels [53], although it is commonly applied for data smoothing in crop phenology monitoring studies [39,63–65].

Optical remote sensing data streams are affected by cloud cover and unfavorable atmospheric conditions, reducing the data temporal continuity [66,67]. Compared to smoothing filters, gap-filling techniques (i.e., fitting functions) allow to reconstruct complete time series with missing data. Given the good precedent of the GPR algorithm as fitting function in reconstructing optically derived LAI time series due to cloud-cover [47,48], the GPR formulation was here adapted as fitting function for gap-filling purposes.

### 2.6 Generation of Green LAI Time Series Collections

Both S2 and L8 GPR LAI models were implemented in an HLS image processing chain for generating LAI time series, which were analyzed for different configurations: S30, L30 and S30+L30. The former two were derived from HLS S2 and L8 surface reflectance dataset, denoted as S30 and L30, respectively. For the latter, previous to the cropland phenology detection, the single-sensor LAI time series derived from each sensor were merged in a single time series by adding up both time series and applying independently and pixel-wise: (1) the local moving Savitzky–Golay smoothing filter and (2) GPR fitting for interpolation and gap-filling processing. These methods were run over all image acquisition dates, leading to continuous time series data streams, hereafter termed as SL30_*SG*_ and SL30_*GPR*_, respectively.

### 2.7 Crop Phenology Estimation

LAI time series smoothing and reconstruction are previous steps to facilitate calculation of phenology metrics [45,68]. The phenology extraction process was conducted for each green LAI time series configuration, over the five years of available time series, focusing on multi-crop parcels, whose major crop types are maize, rice, wheat and clover. Each of the LAI time series collections (S30, L30, SL30_*SG*_, SL30_*GPR*_) were first spatially averaged per parcel and subsequently analyzed to characterize their seasonal dynamics over time by calculating the following key LSP metrics corresponding to crucial stages of crops growth cycle [44]: (1) start-of-season (SOS) or green-up, (2) end-of-season (EOS) or dormancy, (3) length-of-season (LOS)—as the time span in days between SOS and EOS and (4) the seasonal integral area under the curve between SOS and EOS (Area).

The procedure for identifying the number of seasons from a time series is according to previous conventional approaches [69,70]. It makes use of consecutive local minimum, maximum and minimum of the curve to extract a single growing season, when the time series surpasses predefined thresholds. These specific constraints (i.e., thresholds) allow to reduce contributions from undesired artifacts at low frequencies. Consequently, the phenological cycles were evaluated using two criteria [46]: (1) peak prominence and (2) minimum separation thresholds. The former one corresponds to the minimum vertical distance from the season maximum to any side of the peak either reaching a local minimum or an endpoint, i.e., it measures how much the peak stands out due to its intrinsic height and its location relative to other peaks. The latter criteria determines the minimum separation in time between two consecutive potential peaks, so that only the largest maximum within the predefined threshold is selected. Here, the minimum prominence applied ranged from 10% to 30% the difference between the absolute maximum and minimum of a given parcel time series. The minimum separation was set to 90 days. Peaks not meeting the described criteria were discarded.

Regarding the determination of start and end of cropland growth cycles across time, two established strategies were evaluated on their seasonality detection capability, i.e., based on a seasonal or a relative amplitude threshold [44,46,71]:

- Seasonal. Each individual growing season extracted is analyzed to identify key phenological dates (SOS and EOS), when the upward and downward part of the LAI curve defining the growing season reaches a certain percentage fraction of the seasonal amplitude (difference between the maximum and the average of the two local minima per season), respectively.
- Relative. A fixed relative amplitude value is calculated as the difference between the mean maximum and mean minimum of the whole time series and set for all seasons detected, so that the start and end of season occur when the LAI curve reaches a certain percentage fraction of this relative amplitude.

After testing and comparing a range of threshold values on their seasonality detection capability, we defined an optimum threshold of 30% for both methods. Regardless of the seasonality detection approach applied, LOS is calculated as the difference between EOS and SOS. Finally, each season area refers to the integral encompassed between the LAI curve describing a season and the curve minima corresponding to SOS and EOS.

### 2.8 Analysis Setup

First, for each crop parcel and LAI time series collection, the key LSP metrics were extracted from valid growing seasons detected by independently applying the seasonal and relative methods. Then, the corresponding crop type was assigned based on the SOS and EOS dates retrieved throughout the studied years. LSP metrics were statistically analyzed per crop type and year. The availability of a crop planting record for each parcel allowed to assess the growing seasons detection accuracy for each crop type. Finally, spatio-temporal crop seasonality patterns were evaluated over the four years by mapping phenology descriptors over the study site. The smoothing, gap-filling and phenology extraction steps were carried out based on the time series modeling processor imported from the in-house developed scientific toolbox Decomposition and Analysis of Time Series software (DATimeS, v1.12) [46]. DATimeS incorporates conventional interpolation techniques and advanced machine learning fitting algorithms for time series analysis, as well as vegetation phenology modeling methods, with the advantage that unevenly spaced satellite time series can be processed.

## 3 Results

### 3.1 Green LAI Time Series

**Figure 3:**
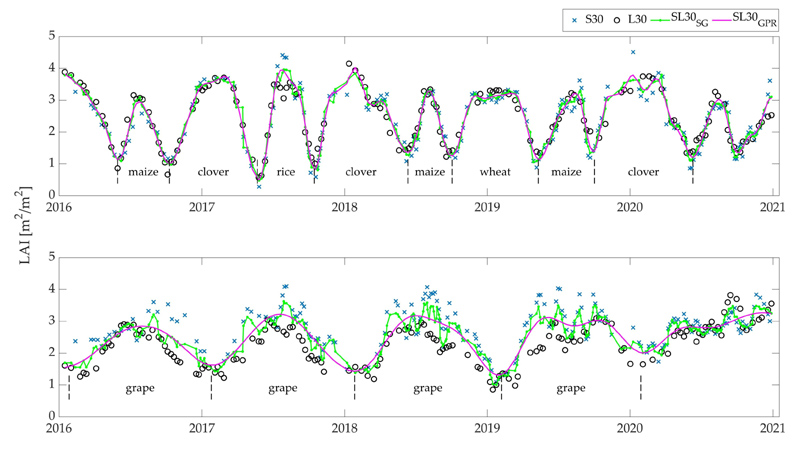
Comparison of S30 and L30 green LAI time series, respectively, derived from S2 and L8 HLS acquisitions and the reconstructed combined LAI time series (SL30_*SG*_ and SL30_*GPR*_) over a multiple (top) and single (bottom) cropping parcel.

The HLS green LAI time series are well able to capture seasonal dynamics from 2016 to the end of 2020 over croplands practicing different cropping systems. See Figure 3 for an illustration. In this example, multi-cropping rotation parcels are characterized by more than one growth cycle per year, with generally two green-up and dormancy events, while in single crop type parcels, the growing season is annually completed. Both cases reflect a good agreement between the two single-sensor LAI estimations, which reveal a relatively high density of cloud-free observations, without large time gaps of missing data, throughout crops growing season. Reconstructed time series derived from the two single-sensor LAI time series provide a more smoothed and temporally continuous LAI profile. This effect is clearly visible over single cropping parcels, which initially present nosier LAI estimations, indicating high spatial heterogeneity and temporal variability.

### 3.2 Crop Phenology Characterization and Evaluation

**Figure 4:**
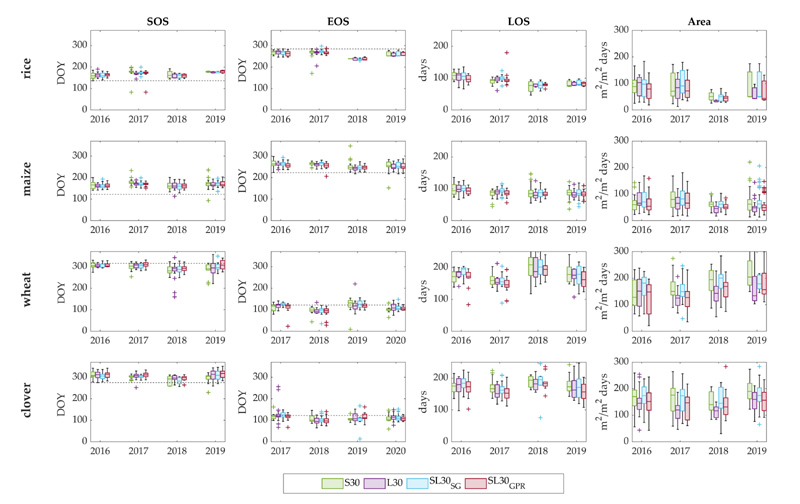
Temporal evolution of LSP metrics distribution of major crops (rice, maize, wheat, clover) parcels per year and time series. Horizontal dashed lines in SOS and EOS boxplots represent planting and harvest dates, respectively.

The temporal evolution of the targeted phenology metrics was computed for the four LAI time series configurations and four crop types. Figure 4 shows the boxplot results of day-of-year (DOY) of the crop phenology metrics along with the planting and harvest dates from the reference crop calendar (Table 1). For later convenience and brevity, only the results obtained from the seasonal method are shown.

Rice and maize SOS were usually detected about 30 days after the planting date. Whereas rice EOS was estimated prior to the harvest date, maize EOS usually fell about 30 days after the harvest. For wheat, SOS was detected more closely to the planting date, and occasionally even before, while EOS was mostly estimated before the harvest date. Summer and winter crops can be clearly differentiated by the LOS values. For the former, the mean value is approximately 90 days, while for the latter it oscillates around 190 days. More than for LOS, a more significant dispersion is shown for Area, suggesting a higher variability in the estimation of this parameter. In general, all time series collections provided similar estimations of the key phenological dates over the four years. Inter-annual variations in phenology are reflected by all LAI time series collections, for instance, the sooner dormancy of rice and maize in 2018, matching the sooner green-up of wheat and clover also reported in 2018.

Table 3 summarizes the global mean and standard deviation of SOS, EOS and LOS for each time series and crop type, comparing the seasonal and relative threshold methods. For the seasonal method, standard deviations for SOS range from 10 to 17 days for summer crops and from 17 to 32 days for wheat and clover. Standard deviation values were analogously registered for the EOS parameter, with a larger variability around the phenological dates detected for winter crops. The range of variation of LOS was also comparable to the previous metrics for summer crops, but not for winter crops, for which the standard deviations were especially higher. Particularly for wheat, a maximum value of 40 days (L30) was obtained.

Results obtained from the relative threshold method show for summer crops similar SOS, EOS and LOS values as the ones from the seasonal method with differences between 1 and 4 days. In the case of winter crops, differences are between 1 and 7 days for SOS and EOS and from 4 to 11 days for LOS. The relative method provided the higher LOS associated standard deviation, up to 53 days (L30), for wheat. In general, for winter crops, the seasonal method estimates green-ups later and dormancies sooner, consequently leading to shorter crop seasons. Considering all crop types together, the seasonal method proved to be able to reduce the overall average standard deviation of SOS and EOS calculated for each of the time series collections. In particular, SL30_*GPR*_ with the seasonal method detected growing seasons across all crop types with a lower overall average standard deviation than the other time series collections, i.e., 15 days for SOS, 16 days for EOS and 22 days for LOS.

The number of growing seasons detected for each crop type and time series configuration is shown in Table 4. As an accuracy assessment, the detection capability was quantified for each crop type by comparing the total number of seasons detected with respect to the reference data of crop fields planted throughout the whole time period studied, expressed as a percentage. From both single-sensor-derived LAI time series, a similar number of phenology cycles were detected for rice and clover using the seasonal method, while for maize and wheat, S30 was able to capture a higher number than L30. The relative method led to a lower number of detected crop seasons for all time series collections and crop types, thus achieving lower detection rates. Particularly with this method, L30 identified more crop seasons over wheat and rice parcels than S30, while S30 identified more crop seasons over maize and clover. Overall, both seasonal and relative detection methods applied to multi-sensor combined time series captured the largest number of growing seasons for all crop types, achieving with GPR-gapfilled LAI time series the highest accuracy with a correct identification of 74% and 66%, respectively. The second best performance of the two methods were obtained with SG filtered time series, with an accuracy of 72% and 65% with the seasonal and relative method, respectively. The seasonal method and GPR are hereafter used for illustrating the subsequent phenology characterization analyzes and cropland mapping carried out.

**Figure 5:**
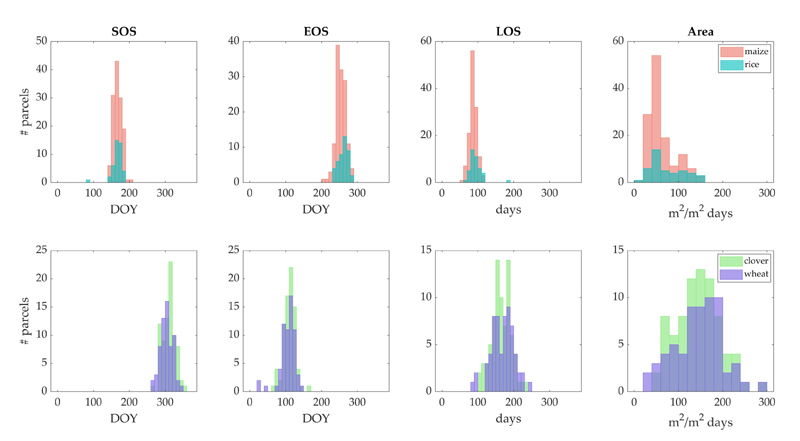
Histogram of SOS, EOS, LOS and Area metrics calculated from SL30_*GPR*_ 2016–2019 time series over all available parcels of maize, rice, clover and wheat crop types. Data are grouped in 15-day bins.

Distinctions in crop seasonal patterns can be observed more precisely from the distribution of phenology metrics over the major crop types parcels shown in the histograms in Figure 5. This figure refers to the four years of SL30_*GPR*_ time series, which were grouped into 15 days bins for SOS, EOS and LOS, while differences of phenology timing between winter and summer crops is apparent, differences in green-up and dormancy dates were not as obvious within crops of the same season. Rice and maize SOS dates follow a close distribution; while rice reaches dormancy later, although over the same time range of distribution as maize. LOS number of days are slightly higher for rice than maize. Likewise, the Area values of both crops present a uniform overlap in the distribution, with relatively higher values for rice than maize. As for winter crops, wheat shows an earlier green-up and a slightly later dormancy than clover, which was already reported in Table 3. Particularly, wheat green-up tends to take place before clover by 15 days ahead. Wheat season length distribution is more irregular than clover, reaching higher values beyond 200 days. Differences in Area are not prominent between the two crop types.

**Figure 6:**
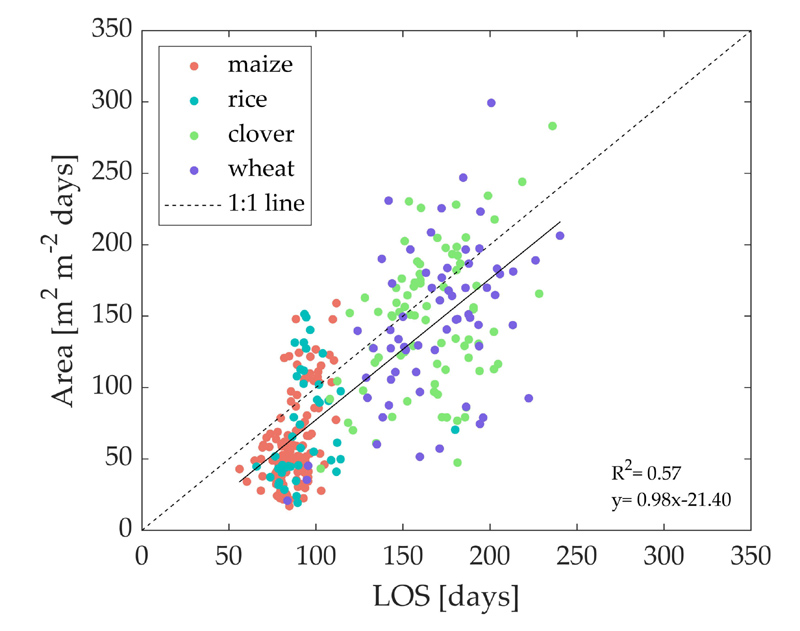
Comparison of Area and LOS for all crop types extracted from 2016 to 2019 SL30_*GPR*_ time series.

A comparison of the LOS against the Area metric is provided in Figure 6. Both parameters show a strong linear correlation with a slope close to 1 (0.98), with higher data pair dispersion for winter crops, with a few LOS outliers values lower than 100 days. In this case, shorter crop seasons are due to an earlier retrieved EOS (Figure 5), while for summer crops, very high LOS values are a consequence of an earlier SOS estimation (Figure 5). All crop types considered, the Area parameter shows a relatively proportional increase regarding the season length. Particularly for summer crops, a remarkable Area variability occurs when approaching the maximum LOS value of 100 days when the highest Area values are also estimated.

### 3.3 Cropping Frequency and Phenology Mapping

**Figure 7:**
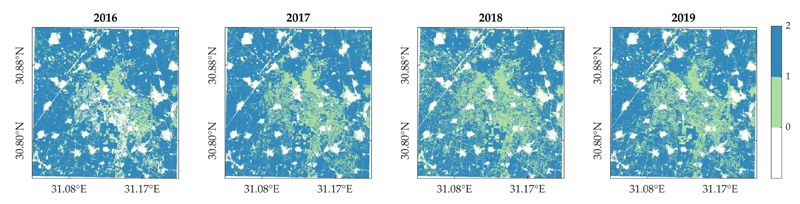
Number of crop growth cycles yearly detected from GPR-gapfilled LAI time series.

The complete HLS LAI time series dataset (S2 and L8) were processed with the GPR interpolation technique with a 10-day regular spacing, as the temporal sampling used for mapping phenology metrics using the seasonal method over the study site. Cropping frequency was mapped as the number of crop growth cycles detected within the period of time studied, allowing to clearly observe the spatial distribution of the two main cropping patterns, for each year from 2016 to 2019 (Figure 7). Multi-cropping parcels being predominant, single-cropping parcels are mainly located in the central region of the study site. The extension covered by each cropping scheme varies locally over the years. Notably, a substantial proportion of pixels located in the center of the study region were not mapped in 2016, as no crop season were detected. Furthermore, when considering all years, the least crop seasons were detected within the multi-cropping area in 2018. This was partly due to the phenology detection method omission inferred from the time series, but mostly because of a delay on cropland seasonality, resulting in a growing winter season computed for the next year, in 2019.

**Figure 8:**
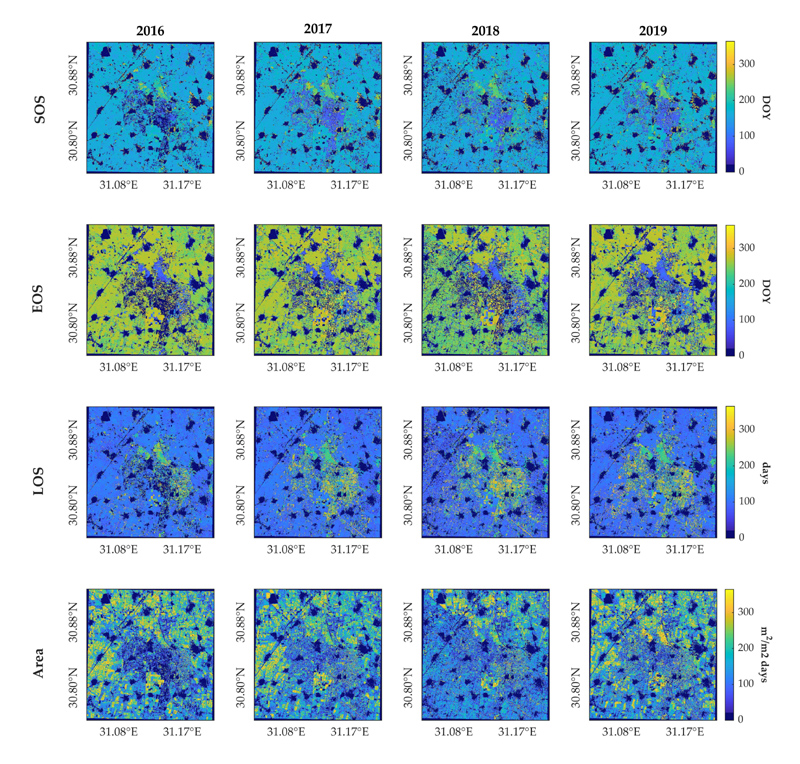
SOS, EOS, LOS and Area maps for the summer season from 2016 to 2019.

**Figure 9:**
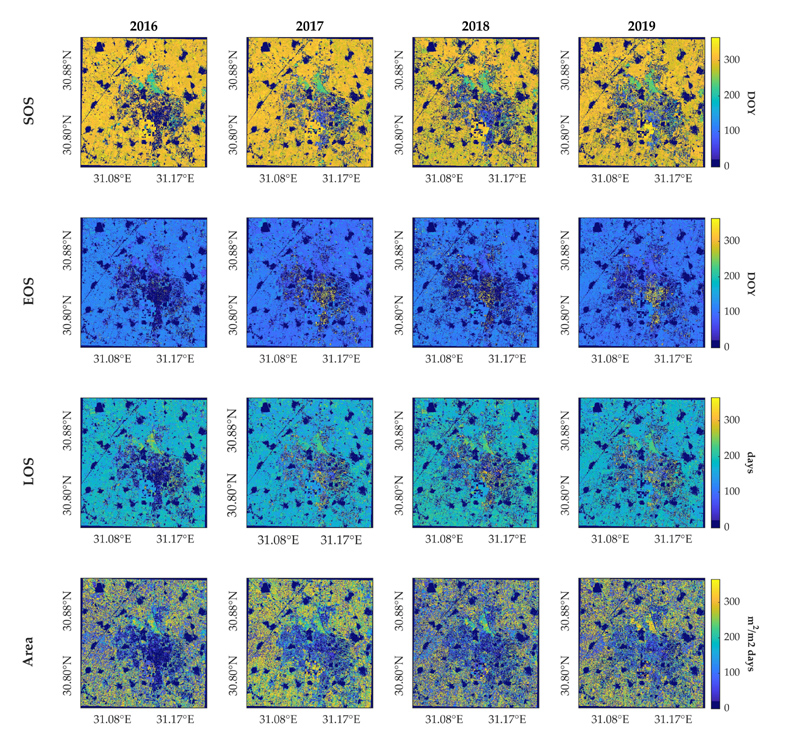
SOS, EOS, LOS and Area maps for the winter season from 2016 to 2019. EOS maps correspond to the following year to SOS.

Cropland phenology temporal evolution of the study site is presented next by distinguishing between summer (Figure 8) and winter crop growing cycles (Figure 9) for the multi-cropping pixels where two crop seasons are identified within a year. It should be noted that while SOS of both summer and winter season crops takes place during the same year, EOS dates corresponding to the latter ones are detected in the next year. Single-cropping pixels with only one growing season identified within a given year are mapped with the same value in the two figures. For better visualization, non-vegetated pixels are masked out applying a maximum value threshold: those pixels whose maximum LAI time series value does not exceed 1 are masked.

LSP metrics of summer season crops (Figure 8) were in general similarly retrieved across the four years. A sooner dormancy occurred in 2018 in accordance with a shorter season length estimation, as well as a notable decrease for Area, shown in Figure 4. Regarding the winter season, although the LSP metrics reveals a similar phenology spatial pattern across the four years, especially in 2016 and 2017, differences can be observed because of an early green-up detected in 2018, shown in Figure 4, consistent with the early dormancy of summer season crops within the same year. Compared to Figure 8, the Area metric exhibits a noisier pattern, though with a regular evolution in time, except in 2017 and 2018, where a general increase and decrease with respect to other years is, respectively, observed.

In both Figures 8 and 9, some pixels were not mapped, since no crop growth cycle was detected within that season year, corresponding to those pixels with a lower number of seasons identified (Figure 7). This was particularly visible in the maps of 2016 and to a lesser extent of 2018. This is caused by intrinsic disturbances in the LAI time series and due to the fact that, occasionally, the time series does not contain the complete growing cycle (see Figure 3), which prevents the phenology method to compute a season.

## 4 Discussion

Having developed a workflow for LSP estimation and cropping patterns mapping from HLS data, the following steps were accomplished: (1) obtaining comparable LAI time series from HLS double-source data streams based on an S2 retrieval model; (2) contrasting the performance of two different threshold-based phenology detection methods; (3) assessing the LSP metrics estimation; and (4) analyzing the time series smoothing and gap-filling methods applied. These steps are further discussed below, followed by encountered limitations and future opportunities of the workflow.

### 4.1 Adaptation of S2 LAI Model to HLS

The availability of harmonized moderate spatial resolution (30 m) satellite products provides a high temporal resolution time series dataset advantageous for cropland monitoring purposes. As opposed to single-source satellite data streams, integration of S2 and L8 increases the temporal frequency of observations, usually leading to improved crop type classification and cropping intensity mapping [50,72]. Moreover, it was previously determined that data from both satellites can provide similar accuracies in LAI retrieval [73]. Furthermore, the HLS two-sensor data collection has shown a superior capability for LAI retrieval as opposed to single-sensor data streams separately in addition to not underestimating high LAI values [74]. The HLS dataset has been previously used for vegetation monitoring, revealing great improvements at detecting phenology at a finer scale from vegetation indices time series, commonly NDVI and EVI2 [21,27,65]. HLS is capable of capturing cropland phenology similarly to higher spatial resolution imagery such as PlanetScope 3 m spatial resolution [75], and is adequate for spatially scaling phenology field observations from GPR-based models [76]. Other multi-source based studies fused HLS data with other satellite data (e.g., Sentinel-1, GOES-16 ABI, VIIRS), allowing high-frequency, quality observations for an improved vegetation phenology estimation [77–79]. Here, four years of an HLS surface reflectance dataset was separately processed into a green LAI time series; while the GPR model was originally trained for estimating LAI from S2 data, the initial GPR model was successfully adapted to the spectral configuration of L8, and showed a compatible and consistent LAI evolution between the different harmonized satellites collections (S30 and L30). Therefore, despite the different original satellites spatial resolution and spectral configuration, our results demonstrated the benefits of the HLS data stream, likely thanks to a common radiometric adjustment and atmospheric correction [26,80].

### 4.2 Comparison of Two Threshold-Based Phenology Detection Methods

The phenology metrics extraction procedure, based on pre-defined amplitude threshold approaches as implemented in DATimeS, allowed to consistently capture cropland seasonal evolution over four consecutive years. Although threshold methods represent the simplest approach to extract phenology metrics from remote sensing time series [10], these methods are easy to tune according to the study location [81] and represent the most common method used to study vegetation dynamics [11]. This study compared two types of thresholds-based approaches, i.e., relative and seasonal amplitude; while the relative amplitude method makes use of a fixed amplitude threshold for the entire time series, the seasonal amplitude method, defined in relative terms, implies an adaptive calculation of phenology timing to individual growing seasons. This approach proved to be more flexible in determining vegetation dynamics over the study area, where typically different crop types are rotated in consecutive years following a periodic scheme. Therefore, each season’s amplitude value varies over time and do not always meet the threshold defined in absolute terms. The relative method has been widely applied for detecting green-up and dormancy events of single growing seasons within a year [16,20,41,82]. Yet, our findings suggest that this threshold is not suitable to be applied for longer term cropland seasonality detection over regions with different inter-annual crop species rotations. However, a quantitative comparison analysis with other well-established phenology detection methods may be necessary to further confirm these findings.

It must also be remarked that SOS and EOS dates, and consequently, the LOS and the Area metrics, can vary depending on the used threshold value, i.e., lowering the threshold generates earlier green-up dates and at the same time the estimation can be affected by noise and small variations in the time series [20]. Usual applied thresholds range from 10% to 50% [11], and may need to be adjusted for each crop type [16,20,71]. With the aim of establishing a standard procedure capable of phenology characterization over single and multi cropping parcels, we proposed a common threshold value of 30%, which allowed to capture as many growing seasons as possible over both types of cropping schemes. Correspondingly, other thresholds (prominence and minimum separation days) were defined as the optimum values in view of the results obtained trying to maximize the number of detected seasons. However, the presented workflow application to monitor larger regions might need another thresholds settings to account for different agroecosystems. Thus, a global parametrization of the threshold-based phenology detection methods should be further investigated.

### 4.3 Evaluation of Crop Phenology Detection

In the absence of in situ crop growth stages data, fixed crop calendar data were used as a reference to evaluate the detection of key phenological events in a general time frame and to additionally assess the suitability of the defined detection threshold. SOS dates were detected after planting and varied within 2–4 weeks, in accordance with previous studies [16,20,43,65], while EOS dates were generally retrieved closer to the harvesting, around 1–3 weeks ahead, except in the case of maize, for which dates were estimated later. Consistency in determining crop phenology was further proven, with a reliable correlation found between two key phenology metrics, such as LOS and Area. It opens opportunities for modeling new agricultural applications in future research, considering, for instance, that the Area parameter can be used to estimate crop yield [83,84]. Furthermore, phenologybased estimation of cropping frequency permitted to monitor the distribution of cropping patterns over the study site. When applied in an operational framework, the derived yearly maps of cropland dynamics can, among others, help assessing if specific crop species rotations affect soil quality [85,86] or the pressure over water resources [51]. Furthermore, characterizing the spatial distribution of different phenological patterns can be useful for distinguishing between vegetation types across heterogeneous landscapes [13,22] in addition to evaluate the impact of climatic changing conditions [50].

### 4.4 High Temporal Resolution for Phenology Detection Improvement

Combined multi-sensor time series were generated using smoothing and gap-filling techniques, obtaining a considerable noise reduction and data continuity, which eventually benefited the automated phenology detection. Each of the analyzed data streams was able to consistently monitor crop progress, with identified similar yearly patterns of crop growth for the same crop type. These patterns varied from year to year, likely due to weather conditions or changes in management practices. No substantial discrepancies in the mean estimates of the LSP metrics were observed between the different LAI time series configurations, mainly due to the initially available number of cloud-free observations from S2 and L8 single-sensor collections over the study site. As such, it permitted to automatically extract crop phenology seasons with a similar timing as the combined reconstructed LAI time series. The results also revealed that combining time series can reduce the dispersion around the estimation of the key crop phenology dates analyzed. More significant is the improvement in the detection of the number of growing seasons, providing S2 an overall accuracy of 69% in comparison to 63% obtained by L8. These results confirm the superior performance of S2 as compared to L8 as also demonstrated in previous studies (see review [49]). The combined time series data flow led to the highest accuracies, with GPR gap-filling being more effective than SG, i.e., 74% and 72%, respectively, in addition to a reduction in the variability of the number of days associated with the key phenological dates.

Given these promising results, the workflow should, however, be extended over other regions where cloud contamination represents a more limiting factor for crop monitoring, so that more substantial improvements derived from multi-sensor data combination can be expected. Furthermore, expected in near future, the recent launch of Landsat 9 (L9) will further contribute to reducing the time gap of data collection, since the combined L8 and L9 revisit time will be each 8 days [87]. In anticipation of a higher frequency of satellite acquisitions, preferences tend to move towards faster time series processing smoothing methods with flexibility to preserve intra-seasonal variations (e.g., Savitzky–Golay [53] and Whittaker [88,89]) [90].

### 4.5 Limitations and Future Opportunities

Cropland monitoring through phenology mapping requires a long-range time series data stream to proper capture crop growing stages, by means of continuous and smooth data. GPR represents an adequate technique for generating continuous time series that can be widely applied without using any ancillary data but with an important inherent disadvantage, i.e., the computational cost. In this respect, some efforts have been made to overcome the computational inconvenience by optimizing the calculation of the GPR hyperparameters per crop type, obtaining a considerable processing time reduction and hardly affecting the fitting accuracy retrieving phenology parameters per pixel, although it is necessary to have a land cover map at disposal [47]. Concerning this constraint, the advent of cloud computing platforms such as Google Earth Engine (GEE) allow to process satellite data for large-scale vegetation mapping with a high-performance computing capacity [91]. With ambition to expand the applicability of this study, the here developed workflow could be potentially implemented into GEE, where a similar GPR LAI retrieval model was recently successfully imported for green LAI mapping from S2 over the Iberian peninsula [48]. Furthermore, image fusion of different satellites (Landsat and MODIS) for time series smoothing and gap-filling has already been investigated [23,92] and a framework for cropland phenology monitoring in GEE using GPR has been recently devised [93]. These works suggest that our proposed workflow can be almost directly integrated into GEE, implying that the phenology detection can be realized anywhere in the world and for any time window. However, some additional efforts will be required to enable integrating the here presented flexible phenology detection method, i.e., with a seasonal-based amplitude threshold. GEE-based phenology studies currently rely on a double logistic model, whereby the SOS and EOS metrics are derived with a half-maximum criterion method, which is unable to distinguish between two or more seasonal cycles [93,94].

## 5 Conclusions

A workflow was developed for cropland phenology estimation based on a green LAI time series derived from NASA’s Harmonized Landsat 8 (L8) and Sentinel-2 (S2) surface reflectance dataset. It was applied over an agricultural region in the Nile Delta from 2016 to 2020. Starting from a green LAI retrieval model previously trained with GPR for S2, an adapted GPR model was trained for L8. Both models were implemented in an image-based retrieval chain, obtaining two different LAI time series data streams, which were used separately and combined to extract LSP metrics for characterizing crop rotation of major cash crops over the study area (maize, rice, wheat, clover). Two time series processing methods were proposed to combine both single-sensor LAI data streams: the (1) the Savitzky–Golay (SG) smoothing filter and (2) the GPR gap-filling fitting technique. Differences in detecting cropland phenology seasonality were compared for the four resulting LAI data streams (i.e., L8, S2 and combined SG and GPR). The evolution of cropland seasonal dynamics were observed by generating yearly phenology maps. Overall, the combined LAI time series data by means of GPR, followed by SG, allowed to detect correctly a higher number of within-year growing seasons per crop type within the same time period, as opposed to the single-sensor LAI time series. Summarizing, this study demonstrated that distinct multiband green LAI retrieval models applied to HLS data can retrieve compatible estimations that were successfully used to reconstruct enhanced time series. A dense temporal image data stream, with a higher cloud-free observations availability provided by L8 with respect to the individually use of S2, proved to be crucial for proper monitoring crop rotations of dynamic and heterogeneous agricultural lands. Although our study focused on the Nile Delta agroecosystem, the proposed workflow is neither site- nor crop-specific, and therefore, can be easily applied over other regions where both S2 and L8 images are available.

## Figures and Tables

**Table 1:** Established crop calendar over the study site (Tanta, El Gharbia governorate, Egypt).

Crop	Planting Date	Harvest Date
rice	15 May	10 October
maize	1 May	10 August
wheat	10 November	1 May
clover	1 October	1 May

**Table 2:** S2 and L8 spectral bands.

	Visible & NIR								SWIR	
S2 band	2	3	4	5	6	7	8	8A	11	12
Wavelength (nm)	490	560	665	705	740	783	842	865	1610	2190
Spatial resolution (m)	10	10	10	20	20	20	10	20	20	20
L8 band	2	3	4	-	-	-	-	5	6	7
Wavelength (nm)	480	560	655	–	-	–	–	865	1610	2200
Spatial resolution (m)	30	30	30	-	-	-	-	30	30	30

**Table 3:** Mean value and standard deviation (SD) in days of the key LSP metrics (SOS (DOY), EOS (DOY) and LOS (days)) per crop type and time series collection, comparing the two phenology detection methods (sesonal and relative). An overall standard deviation average in days was calculated from all crop types and each time series collection.

Time series	Rice					
	$\bar{SOS}$ ± SD		$\bar{EOS}$ ± SD		$\bar{LOS}$ ± SD	
	Seasonal	Relative	Seasonal	Relative	Seasonal	Relative
S30	171 ± 16	173 ± 17	265 ± 13	263 ± 14	94 ± 17	90 ± 21
L30	165 ± 10	161 ± 21	264 ± 13	263 ± 14	99 ± 14	102 ± 24
SL30_*SG*_	166 ± 12	166 ± 19	264 ± 15	265 ± 13	98 ± 17	98 ± 22
SL30_*GPR*_	166 ± 16	170 ± 19	260 ± 13	260 ± 15	94 ± 18	90 ± 24
	**Maize**					
	$\bar{SOS}$ ± SD		$\bar{EOS}$ ± SD		$\bar{LOS}$ ± SD	
	Seasonal	Relative	Seasonal	Relative	Seasonal	Relative
S30	169 ± 17	169 ± 15	259 ± 15	256 ± 19	90 ± 17	87 ± 18
L30	165 ± 12	165 ± 12	254 ± 14	252 ± 16	89 ± 14	87 ± 18
SL30_*SG*_	166 ± 13	167 ± 13	258 ± 15	255 ± 18	92 ± 15	89 ± 17
SL30_*GPR*_	167 ± 11	168 ± 11	254 ± 14	253 ± 16	87 ± 10	85 ± 15
	**Wheat**					
	$\bar{SOS}$ ± SD		$\bar{EOS}$ ± SD		$\bar{LOS}$ ± SD	
	Seasonal	Relative	Seasonal	Relative	Seasonal	Relative
S30	294 ± 23	290 ± 30	110 ± 24	116 ± 29	181 ± 34	192 ± 45
L30	294 ± 32	290 ± 39	108 ± 18	109 ± 27	179±40	184±53
SL30_*SG*_	298 ± 17	295 ± 19	113 ± 21	114±23	180 ± 33	184±36
SL30_*GPR*_	303 ± 17	296 ± 28	106 ± 22	107 ± 24	168 ± 32	177 ± 40
	**Clover**					
	$\bar{SOS}$ ± SD		$\bar{EOS}$ ± SD		$\bar{LOS}$ ± SD	
	Seasonal	Relative	Seasonal	Relative	Seasonal	Relative
S30	302 ± 19	298 ± 26	110 ± 17	113±21	173±24	180 ± 34
L30	307 ± 18	301 ± 24	112 ± 25	117±26	170 ± 33	181 ± 37
SL30_*SG*_	303 ± 18	300 ± 16	113 ± 21	118 ± 19	176 ± 30	184±28
SL30_*GPR*_	310 ± 17	305 ± 18	110 ± 16	114±16	165±27	174±25
	**Total**					
	$\bar{SD}$ _*SOS*_		$\bar{SD}$ _*EOS*_		$\bar{SD}$ _*LOS*_	
	Seasonal	Relative	Seasonal	Relative	Seasonal	Relative
S30	19	22	17	21	23	29
L30	18	24	17	21	25	33
SL30_*SG*_	15	17	18	18	24	26
SL30_*GPR*_	15	19	16	18	22	26

**Table 4:** Number (#) and accuracy detection (%) of crop seasons with respect of the number of crops planted per time series collection and crop type from 2016 to 2019 over all available parcels. The highest accuracy (%) per crop type and method columns is bolded.

Time Series	Rice				Maize				Wheat				Clover				Total			
	Seasonal		Relative		Seasonal		Relative		Seasonal		Relative		Seasonal		Relative		Seasonal		Relative	
	#	%	#	%	#	%	#	%	#	%	#	%	#	%	#	%	#	%	#	%
S30	36	60	33	55	124	71	105	60	60	67	52	58	73	72	66	65	293	69	256	60
L30	36	60	36	60	110	63	99	57	55	61	57	**63**	69	68	63	62	270	63	255	60
SL30_*SG*_	42	**70**	37	62	131	**75**	118	**67**	58	64	52	58	75	74	71	**70**	306	72	278	65
SL30_*GPR*_	42	**70**	39	**65**	131	**75**	115	66	63	**70**	56	62	79	**77**	70	69	315	**74**	280	**66**

## References

[1] Elmore AJ, Guinn SM, Minsley BJ, Richardson AD (2012). Landscape controls on the timing of spring, autumn, and growing season length in mid-A tlantic forests. Glob Chang Biol.

[2] Estel S, Kuemmerle T, Levers C, Baumann M, Hostert P (2016). Mapping cropland-use intensity across Europe using MODIS NDVI time series. Environ Res Lett.

[3] Moon M, Li D, Liao W, Rigden AJ, Friedl MA (2020). Modification of surface energy balance during springtime: The relative importance of biophysical and meteorological changes. Agric For Meteorol.

[4] Atzberger C (2013). Advances in Remote Sensing of Agriculture: Context Description, Existing Operational Monitoring Systems and Major Information Needs. Remote Sens.

[5] Richardson AD, Keenan TF, Migliavacca M, Ryu Y, Sonnentag O, Toomey M (2013). Climate change, phenology, and phenological control of vegetation feedbacks to the climate system. Agric For Meteorol.

[6] Weiss M, Jacob F, Duveiller G (2020). Remote sensing for agricultural applications: A meta-review. Remote Sens Environ.

[7] Ray DK, Mueller ND, West PC, Foley JA (2013). Yield trends are insufficient to double global crop production by 2050. PLoS ONE.

[8] Tilman D, Balzer C, Hill J, Befort BL (2011). Global food demand and the sustainable intensification of agriculture. Proc Natl Acad Sci USA.

[9] Wu W, Yu Q, You L, Chen K, Tang H, Liu J (2018). Global cropping intensity gaps: Increasing food production without cropland expansion. Land Use Policy.

[10] Zeng L, Wardlow BD, Xiang D, Hu S, Li D (2020). A review of vegetation phenological metrics extraction using time-series, multispectral satellite data. Remote Sens Environ.

[11] Caparros-Santiago JA, Rodriguez-Galiano V, Dash J (2021). Land surface phenology as indicator of global terrestrial ecosystem dynamics: A systematic review. ISPRS J Photogramm Remote Sens.

[12] Liu J, Zhu W, Atzberger C, Zhao A, Pan Y, Huang X (2018). A phenology-based method to map cropping patterns under a wheat-maize rotation using remotely sensed time-series data. Remote Sens.

[13] Hu Q, Sulla-Menashe D, Xu B, Yin H, Tang H, Yang P, Wu W (2019). A phenology-based spectral and temporal feature selection method for crop mapping from satellite time series. Int J Appl Earth Obs Geoinf.

[14] Hill MJ, Donald GE (2003). Estimating spatio-temporal patterns of agricultural productivity in fragmented landscapes using AVHRR NDVI time series. Remote Sens Environ.

[15] Delbart N, Le Toan T, Kergoat L, Fedotova V (2006). Remote sensing of spring phenology in boreal regions: A free of snow-effect method using NOAA-AVHRR and SPOT-VGT data (1982-2004). Remote Sens Environ.

[16] Liu L, Zhang X, Yu Y, Gao F, Yang Z (2018). Real-time monitoring of crop phenology in the Midwestern United States using VIIRS observations. Remote Sens.

[17] Zhang X, Liu L, Liu Y, Jayavelu S, Wang J, Moon M, Henebry GM, Friedl MA, Schaaf CB (2018). Generation and evaluation of the VIIRS land surface phenology product. Remote Sens Environ.

[18] Panday US, Pratihast AK, Aryal J, Kayastha RB (2020). A review on drone-based data solutions for cereal crops. Drones.

[19] Fisher JI, Mustard JF, Vadeboncoeur MA (2006). Green leaf phenology at Landsat resolution: Scaling from the field to the satellite. Remote Sens Environ.

[20] Gao F, Anderson MC, Zhang X, Yang Z, Alfieri JG, Kustas WP, Mueller R, Johnson DM, Prueger JH (2017). Toward mapping crop progress at field scales through fusion of Landsat and MODIS imagery. Remote Sens Environ.

[21] Zhang X, Wang J, Henebry GM, Gao F (2020). Development and evaluation of a new algorithm for detecting 30 m land surface phenology from VIIRS and HLS time series. ISPRS J Photogramm Remote Sens.

[22] Li R, Xu M, Chen Z, Gao B, Cai J, Shen F, He X, Zhuang Y, Chen D (2021). Phenology-based classification of crop species and rotation types using fused MODIS and Landsat data: The comparison of a random-forest-based model and a decision-rule-based model. Soil Tillage Res.

[23] Nietupski TC, Kennedy RE, Temesgen H, Kerns BK (2021). Spatiotemporal image fusion in Google Earth Engine for annual estimates of land surface phenology in a heterogenous landscape. Int J Appl Earth Obs Geoinf.

[24] Gao F, Anderson MC, Johnson DM, Seffrin R, Wardlow B, Suyker A, Diao C, Browning DM (2021). Towards Routine Mapping of Crop Emergence within the Season Using the Harmonized Landsat and Sentinel-2 Dataset. Remote Sens.

[25] ED Chaves M, CA Picoli M, D Sanches I (2020). Recent Applications of Landsat 8/OLI and Sentinel-2/MSI for Land Use and Land Cover Mapping: A Systematic Review. Remote Sens.

[26] Claverie M, Ju J, Masek JG, Dungan JL, Vermote EF, Roger JC, Skakun SV, Justice C (2018). The Harmonized Landsat and Sentinel-2 surface reflectance data set. Remote Sens Environ.

[27] Bolton DK, Gray JM, Melaas EK, Moon M, Eklundh L, Friedl MA (2020). Continental-scale land surface phenology from harmonized Landsat 8 and Sentinel-2 imagery. Remote Sens Environ.

[28] Zhou Q, Rover J, Brown J, Worstell B, Howard D, Wu Z, Gallant AL, Rundquist B, Burke M (2019). Monitoring landscape dynamics in central us grasslands with harmonized Landsat-8 and Sentinel-2 time series data. Remote Sens.

[29] Hao P, Tang H, Chen Z, Le Y, Wu M (2019). High resolution crop intensity mapping using harmonized Landsat-8 and Sentinel-2 data. J Integr Agric.

[30] Wang S, Zhang L, Huang C, Qiao N (2017). An NDVI-based vegetation phenology is improved to be more consistent with photosynthesis dynamics through applying a light use efficiency model over boreal high-latitude forests. Remote Sens.

[31] Gitelson AA (2004). Wide dynamic range vegetation index for remote quantification of biophysical characteristics of vegetation. J Plant Physiol.

[32] Sakamoto T, Wardlow BD, Gitelson AA, Verma SB, Suyker AE, Arkebauer TJ (2010). A two-step filtering approach for detecting maize and soybean phenology with time-series MODIS data. Remote Sens Environ.

[33] Yan G, Hu R, Luo J, Weiss M, Jiang H, Mu X, Xie D, Zhang W (2019). Review of indirect optical measurements of leaf area index: Recent advances, challenges, and perspectives. Agric For Meteorol.

[34] Liu J, Pattey E, Jégo G (2012). Assessment of vegetation indices for regional crop green LAI estimation from Landsat images over multiple growing seasons. Remote Sens Environ.

[35] Pasqualotto N, Delegido J, Van Wittenberghe S, Rinaldi M, Moreno J (2019). Multi-crop green LAI estimation with a new simple Sentinel-2 LAI Index (SeLI). Sensors.

[36] Verrelst J, Malenovsky Z, Van der Tol C, Camps-Valls G, Gastellu-Etchegorry JP, Lewis P, North P, Moreno J (2019). Quantifying vegetation biophysical variables from imaging spectroscopy data: A review on retrieval methods. Surv Geophys.

[37] Fang H, Baret F, Plummer S, Schaepman-Strub G (2019). An overview of global leaf area index (LAI): Methods, products, validation, and applications. Rev Geophys.

[38] Verger A, Filella I, Baret F, Peñuelas J (2016). Vegetation baseline phenology from kilometric global LAI satellite products. Remote Sens Environ.

[39] Wang C, Zhang Z, Chen Y, Tao F, Zhang J, Zhang W (2018). Comparing different smoothing methods to detect double-cropping rice phenology based on LAI products–a case study in the Hunan province of China. Int J Remote Sens.

[40] Wang C, Li J, Liu Q, Zhong B, Wu S, Xia C (2017). Analysis of differences in phenology extracted from the enhanced vegetation index and the leaf area index. Sensors.

[41] Diao C (2020). Remote sensing phenological monitoring framework to characterize corn and soybean physiological growing stages. Remote Sens Environ.

[42] Li X, Zhou Y, Asrar GR, Meng L (2017). Characterizing spatiotemporal dynamics in phenology of urban ecosystems based on Landsat data. Sci Total Environ.

[43] Sakamoto T (2018). Refined shape model fitting methods for detecting various types of phenological information on major US crops. ISPRS J Photogramm Remote Sens.

[44] Jönsson P, Eklundh L (2004). TIMESAT—A program for analyzing time-series of satellite sensor data. Comput Geosci.

[45] Kandasamy S, Baret F, Verger A, Neveux P, Weiss M (2013). A comparison of methods for smoothing and gap filling time series of remote sensing observations–application to MODIS LAI products. Biogeosciences.

[46] Belda S, Pipia L, Morcillo-Pallarés P, Rivera-Caicedo JP, Amin E, De Grave C, Verrelst J (2020). DATimeS: A machine learning time series GUI toolbox for gap-filling and vegetation phenology trends detection. Environ Model Softw.

[47] Belda S, Pipia L, Morcillo-Pallarés P, Verrelst J (2020). Optimizing Gaussian Process Regression for Image Time Series Gap-Filling and Crop Monitoring. Agronomy.

[48] Pipia L, Amin E, Belda S, Salinero-Delgado M, Verrelst J (2021). Green LAI Mapping and Cloud Gap-Filling Using Gaussian Process Regression in Google Earth Engine. Remote Sens.

[49] Misra G, Cawkwell F, Wingler A (2020). Status of phenological research using Sentinel-2 data: A review. Remote Sens.

[50] Liu L, Xiao X, Qin Y, Wang J, Xu X, Hu Y, Qiao Z (2020). Mapping cropping intensity in China using time series Landsat and Sentinel-2 images and Google Earth Engine. Remote Sens Environ.

[51] Biradar CM, Xiao X (2011). Quantifying the area and spatial distribution of double-and triple-cropping croplands in India with multi-temporal MODIS imagery in 2005. Int J Remote Sens.

[52] Amin E, Verrelst J, Rivera-Caicedo JP, Pipia L, Ruiz-Verdú A, Moreno J (2021). Prototyping Sentinel-2 green LAI and brown LAI products for cropland monitoring. Remote Sens Environ.

[53] Press WH, Teukolsky SA (1990). Savitzky-Golay smoothing filters. Comput Phys.

[54] Camps-Valls G, Verrelst J, Munoz-Mari J, Laparra V, Mateo-Jimenez F, Gomez-Dans J (2016). A survey on Gaussian processes for earth-observation data analysis: A comprehensive investigation. IEEE Geosci Remote Sens Mag.

[55] Eklundh L, Jönsson P (2017). TIMESAT 3.3 with Seasonal Trend Decomposition and Parallel Processing Software Manual.

[56] Vermote E, Roger JC, Franch B, Skakun S LaSRC (Land Surface Reflectance Code): Overview, application and validation using MODIS, VIIRS, LANDSAT and Sentinel 2 data’s.

[57] Verrelst J, Rivera JP, Veroustraete F, Muñoz-Marí J, Clevers JG, Camps-Valls G, Moreno J (2015). Experimental Sentinel-2 LAI estimation using parametric, non-parametric and physical retrieval methods—A comparison. ISPRS J Photogramm Remote Sens.

[58] Verrelst J, Rivera J, Moreno J, Camps-Valls G (2013). Gaussian processes uncertainty estimates in experimental Sentinel-2 LAI and leaf chlorophyll content retrieval. ISPRS J Photogramm Remote Sens.

[59] Verrelst J, Rivera JP, Gitelson A, Delegido J, Moreno J, Camps-Valls G (2016). Spectral band selection for vegetation properties retrieval using Gaussian processes regression. Int J Appl Earth Obs Geoinf.

[60] Drusch M, Del Bello U, Carlier S, Colin O, Fernandez V, Gascon F, Hoersch B, Isola C, Laberinti P, Martimort P (2012). Sentinel-2: ESA’s Optical High-Resolution Mission for GMES Operational Services. Remote Sens Environ.

[61] Rasmussen C, Williams C (2006). Gaussian Processes for Machine Learning.

[62] Blum M, Riedmiller M Optimization of Gaussian Process Hyperparameters using Rprop.

[63] Chen J, Jönsson P, Tamura M, Gu Z, Matsushita B, Eklundh L (2004). A simple method for reconstructing a high-quality NDVI time-series data set based on the Savitzky–Golay filter. Remote Sens Environ.

[64] Pan Z, Huang J, Zhou Q, Wang L, Cheng Y, Zhang H, Blackburn GA, Yan J, Liu J (2015). Mapping crop phenology using NDVI time-series derived from HJ-1 A/B data. Int J Appl Earth Obs Geoinf.

[65] Gao F, Anderson M, Daughtry C, Karnieli A, Hively D, Kustas W (2020). A within-season approach for detecting early growth stages in corn and soybean using high temporal and spatial resolution imagery. Remote Sens Environ.

[66] Weiss DJ, Atkinson PM, Bhatt S, Mappin B, Hay SI, Gething PW (2014). An effective approach for gap-filling continental scale remotely sensed time-series. ISPRS J Photogramm Remote Sens.

[67] Zhou J, Jia L, Menenti M, Gorte B (2016). On the performance of remote sensing time series reconstruction methods–A spatial comparison. Remote Sens Environ.

[68] Atkinson PM, Jeganathan C, Dash J, Atzberger C (2012). Inter-comparison of four models for smoothing satellite sensor time-series data to estimate vegetation phenology. Remote Sens Environ.

[69] Lloyd D (1990). A phenological classification of terrestrial vegetation cover using shortwave vegetation index imagery. Int J Remote Sens.

[70] White MA, Nemani RR (2006). Real-time monitoring and short-term forecasting of land surface phenology. Remote Sens Environ.

[71] Huang X, Liu J, Zhu W, Atzberger C, Liu Q (2019). The Optimal Threshold and Vegetation Index Time Series for Retrieving Crop Phenology Based on a Modified Dynamic Threshold Method. Remote Sens.

[72] Tian H, Huang N, Niu Z, Qin Y, Pei J, Wang J (2019). Mapping winter crops in China with multi-source satellite imagery and phenology-based algorithm. Remote Sens.

[73] Meyer LH, Heurich M, Beudert B, Premier J, Pflugmacher D (2019). Comparison of Landsat-8 and Sentinel-2 data for estimation of leaf area index in temperate forests. Remote Sens.

[74] Mourad R, Jaafar H, Anderson M, Gao F (2020). Assessment of leaf area index models using harmonized landsat and sentinel-2 surface reflectance data over a semi-arid irrigated landscape. Remote Sens.

[75] Moon M, Richardson AD, Friedl MA (2021). Multiscale assessment of land surface phenology from harmonized Landsat 8 and Sentinel-2, PlanetScope, and PhenoCam imagery. Remote Sens Environ.

[76] Burke MW, Rundquist BC (2021). Scaling Phenocam GCC, NDVI, and EVI2 with Harmonized Landsat-Sentinel using Gaussian Processes. Agric For Meteorol.

[77] Torbick N, Huang X, Ziniti B, Johnson D, Masek J, Reba M (2018). Fusion of moderate resolution earth observations for operational crop type mapping. Remote Sens.

[78] Shen Y, Zhang X, Wang W, Nemani R, Ye Y, Wang J (2021). Fusing Geostationary Satellite Observations with Harmonized Landsat-8 and Sentinel-2 Time Series for Monitoring Field-Scale Land Surface Phenology. Remote Sens.

[79] Shen Y, Zhang X, Yang Z (2022). Mapping corn and soybean phenometrics at field scales over the United States Corn Belt by fusing time series of Landsat 8 and Sentinel-2 data with VIIRS data. ISPRS J Photogramm Remote Sens.

[80] Pastick NJ, Wylie BK, Wu Z (2018). Spatiotemporal analysis of Landsat-8 and Sentinel-2 data to support monitoring of dryland ecosystems. Remote Sens.

[81] Tan B, Morisette JT, Wolfe RE, Gao F, Ederer GA, Nightingale J, Pedelty JA (2010). An enhanced TIMESAT algorithm for estimating vegetation phenology metrics from MODIS data. IEEE J Sel Top Appl Earth Obs Remote Sens.

[82] Moon M, Zhang X, Henebry GM, Liu L, Gray JM, Melaas EK, Friedl MA (2019). Long-term continuity in land surface phenology measurements: a comparative assessment of the MODIS land cover dynamics and VIIRS land surface phenology products. Remote Sens Environ.

[83] Franch B, Vermote EF, Skakun S, Roger JC, Becker-Reshef I, Murphy E, Justice C (2019). Remote sensing based yield monitoring: Application to winter wheat in United States and Ukraine. Int J Appl Earth Obs Geoinf.

[84] Skakun S, Vermote E, Franch B, Roger JC, Kussul N, Ju J, Masek J (2019). Winter wheat yield assessment from Landsat 8 and Sentinel-2 data: Incorporating surface reflectance, through phenological fitting, into regression yield models. Remote Sens.

[85] Congreves K, Hayes A, Verhallen E, Van Eerd L (2015). Long-term impact of tillage and crop rotation on soil health at four temperate agroecosystems. Soil Tillage Res.

[86] Triberti L, Nastri A, Baldoni G (2016). Long-term effects of crop rotation, manure and mineral fertilisation on carbon sequestration and soil fertility. Eur J Agron.

[87] Masek JG, Wulder MA, Markham B, McCorkel J, Crawford CJ, Storey J, Jenstrom DT (2020). Landsat 9: Empowering open science and applications through continuity. Remote Sens Environ.

[88] Eilers PH (2003). A perfect smoother. Anal Chem.

[89] Atzberger C, Eilers PH (2011). A time series for monitoring vegetation activity and phenology at 10-daily time steps covering large parts of South America. Int J Digit. Earth.

[90] Jönsson P, Cai Z, Melaas E, Friedl MA, Eklundh L (2018). A method for robust estimation of vegetation seasonality from Landsat and Sentinel-2 time series data. Remote Sens.

[91] Gorelick N, Hancher M, Dixon M, Ilyushchenko S, Thau D, Moore R (2017). Google Earth Engine: Planetary-scale geospatial analysis for everyone. Remote Sens Environ.

[92] Moreno-Martínez Á, Izquierdo-Verdiguier E, Maneta MP, Camps-Valls G, Robinson N, Muñoz-Marí J, Sedano F, Clinton N, Running SW (2020). Multispectral high resolution sensor fusion for smoothing and gap-filling in the cloud. Remote Sens Environ.

[93] Salinero-Delgado M, Estévez J, Pipia L, Belda S, Berger K, Paredes Gómez V, Verrelst J (2022). Monitoring Cropland Phenology on Google Earth Engine Using Gaussian Process Regression. Remote Sens.

[94] Li X, Zhou Y, Meng L, Asrar GR, Lu C, Wu Q (2019). A dataset of 30 m annual vegetation phenology indicators (1985-2015) in urban areas of the conterminous United States. Earth Syst Sci Data.

